# Concordance of rapid whole genome and targeted long read sequencing of pediatric brain tumors with standard clinical testing

**DOI:** 10.21203/rs.3.rs-10885746/v1

**Published:** 2026-09-02

**Authors:** Min Seon Park, Erin Crotty, Trent Prall, Miranda PG Zalusky, Jeff Stevens, Vera Paulson, Rebecca Ronsley, Kristyn Galbraith, Joy Goffena, Sophie HR Storz, Zachery B. Anderson, Angela L. Miller, Matthew Snyder, Bonnie Cole, Sarah Leary, Tina Lockwood, Danny Miller

**Affiliations:** University of Washington; Seattle Children’s Hospital; University of Washington; University of Washington; Seattle Children’s Research Institute; University of Washington; Fred Hutch Cancer Center; University of Washington; University of Washington; University of Washington; University of Washington; University of Washington; University of Washington; Seattle Children’s Hospital; Seattle Children’s Hospital; University of Washington; University of Washington

**Keywords:** long-read sequencing, nanopore sequencing, pediatric brain tumors, DNA methylation classification, adaptive sampling, molecular diagnostics

## Abstract

**Background:**

The 2021 WHO classification of central nervous system tumors requires integrated molecular testing for accurate diagnosis and risk stratification. Current clinical workflows typically require separate assays for single nucleotide variants (SNVs), copy number variants (CNVs), structural variants (SVs), and DNA methylation profiling, leading to diagnostic delays of weeks to months and substantial tissue consumption. Long-read sequencing (LRS) offers the potential to deliver all required molecular data from a single assay with dramatically reduced turnaround time.

**Methods:**

We evaluated 23 pediatric brain tumor samples, representing 10 different tumor types, using LRS on the Oxford Nanopore Technologies (ONT) platform, performing whole-genome sequencing (wgLRS; *n*=9), adaptive sampling (AS; *n*=20), or both (*n*=6). Using a streamlined protocol with an approximately 60-minute sample preparation time, we compared LRS-derived variant calls and methylation-based tumor classification against clinical testing results.

**Results:**

Both wgLRS and AS showed high concordance with clinical testing across all variant classes. SNVs and indels were detected with high sensitivity above 15% allele fraction (42 of 51 clinically reported variants), and a somatic-optimized caller recovered four additional driver mutations missed by the standard germline pipeline. CNV profiles showed strong agreement with clinical testing, and 5 of 6 clinically reported gene fusions were identified. Methylation classification was concordant with clinical diagnosis in 18 of 22 (82%) evaluable tumors after only 10 minutes of sequencing using Sturgeon alone, increasing to 19 of 22 (86%) within one hour of sequencing using three real-time classifiers. Adding MNP-Flex, which draws on an expanded 184-class reference, resolved one additional tumor for an overall methylation concordance of 20 of 22 (91%). All discordances were attributable to differences in sampled tissue (autopsy versus surgical specimen), low tumor content, subclonal variants below detection thresholds, or limitations of classifier reference sets.

**Conclusion:**

LRS allows for comprehensive molecular characterization of pediatric brain tumors from a single library preparation, with methylation-based classification often available within an hour of sequencing initiation. This integrated approach has the potential to accelerate diagnostic workflows, reduce tissue requirements, tailor neurosurgical approaches, and facilitate timely treatment decisions in pediatric neuro-oncology.

## INTRODUCTION

Pediatric brain tumors are the leading cause of cancer-related mortality in children, with over 4,000 new cases diagnosed annually in the United States.^1^ The 2021 World Health Organization (WHO) Classification of Tumors of the Central Nervous System fundamentally transformed diagnostic practice by necessitating integrated molecular characterization alongside histopathological assessment.^2^ Under this framework, accurate diagnosis and risk stratification depend on the identification of defining genetic alterations—including single nucleotide variants (SNVs), copy number variants (CNVs), structural variants (SVs), and gene fusions—as well as DNA methylation-based tumor classification.^3^ This molecular complexity creates substantial diagnostic challenges and delays, particularly for pediatric patients where timely initiation of appropriate therapy is critical.^4^

Current clinical workflows address these molecular requirements through a fragmented, multi-platform testing approach. Comprehensive characterization of a pediatric brain tumor may require targeted sequencing panels for SNV and indel detection, chromosomal microarray analysis or shallow whole-genome sequencing for CNV profiling, RNA sequencing or fusion panels for SV identification, and methylation arrays for epigenetic classification (Figure 1A).^3,5^ Each assay consumes limited tissue, requires separate laboratory workflows, and adds days to weeks to the diagnostic timeline. For institutions without molecular testing capabilities, samples must be sent to reference laboratories, further extending turnaround times. The cumulative effect is that definitive molecular diagnosis may lag weeks to months behind initial histopathological assessment, delaying treatment decisions for individuals with aggressive malignancies.

Tissue limitations inherent to pediatric brain tumor diagnosis compound these challenges. Surgical biopsies, particularly from deep-seated locations, often yield minimal material. When this limited tissue must be divided among multiple testing platforms, individual assays may have insufficient input, potentially compromising sensitivity or precluding testing altogether. A diagnostic approach that could derive comprehensive molecular information from a single aliquot of tumor tissue would represent a substantial advance.

Long-read sequencing (LRS) technologies offer a potential solution to these diagnostic bottlenecks. Unlike short-read sequencing, which generates reads of 150–300 base pairs, LRS produces reads spanning thousands to tens of thousands of base pairs.^6^ This read length confers several analytical advantages relevant to brain tumor diagnosis: improved mapping in repetitive genomic regions, direct detection of SVs and gene fusions without inference from discordant read pairs, and native identification of DNA methylation without bisulfite conversion (Figure 1B).^7,8^ Critically, sequencing on the Oxford Nanopore Technologies (ONT) platform enables real-time data analysis, allowing preliminary results to be evaluated while sequencing is still in progress.^9^ In addition, ONT adaptive sampling (AS) is a computational target enrichment approach that selectively sequences pre-defined genomic regions of interest by ejecting off-target reads in real time. This approach can achieve high depth of coverage in clinically relevant genes while also retaining genome-wide background reads sufficient for copy number analysis and methylation profiling.^10,11^

Several recent studies have demonstrated the potential of ONT-based LRS for brain tumor molecular profiling. Real-time methylation classification using ONT has been shown to achieve accurate tumor typing within minutes to hours of run initiation, with multiple classifier tools now available, including Sturgeon,^12^ Rapid-CNS^2^,^11^ and nanoDx.^13^ These studies demonstrated the potential of intraoperative ONT-based methylation classification in both adult and pediatric patients,^14,15^ and a recent multicenter validation of Rapid-CNS^2^ demonstrated methylation classification within a 30-minute intraoperative window followed by comprehensive molecular profiling within 24 hours.^16^ Workflows integrating methylation classification with SNV, CNV, and SV detection from a single ONT assay have been described,^11,17^ and the ROBIN platform recently demonstrated intraoperative classification with next-day full molecular profiling in 50 prospective cases.^17^ However, these studies have predominantly evaluated adult gliomas or mixed adult-pediatric cohorts, have often assessed only subsets of the required molecular characterization, or have used relatively small numbers of pediatric cases that do not capture the full diversity of pediatric CNS tumor types. Furthermore, the relative performance of whole-genome LRS (wgLRS) versus AS approaches for clinical brain tumor profiling has not been systematically compared.

Here, we report a systematic concordance analysis of wgLRS and AS applied to 23 pediatric brain tumor samples representing the major tumor types encountered in clinical practice, including medulloblastoma, high-grade glioma and diffuse midline glioma, ependymoma, atypical teratoid/rhabdoid tumor, choroid plexus carcinoma, embryonal tumor with multilayered rosettes, and pineoblastoma (Figure 1C). We compare LRS-derived variant calls and methylation-based classification against prior clinical testing results across all variant classes, evaluate the speed of real-time methylation classification, and directly compare the diagnostic yield of wgLRS and AS approaches. We demonstrate that LRS achieves high concordance with multi-platform clinical testing, with accurate methylation-based classification available within the first hour of sequencing in most cases using both wgLRS and AS. We further show that a somatic-optimized variant calling pipeline recovers clinically significant mutations. Together, our results demonstrate that LRS-based evaluation of pediatric brain tumors is highly concordant with standard clinical molecular testing and provides complementary information.

## RESULTS

### Study Cohort and Sequencing Approach

We evaluated 23 pediatric brain tumor samples collected at Seattle Children’s Hospital between January 2024 and July 2025 (Table 1, **Supplementary Table 1**). For narrative clarity, individual patient cases are referred to throughout by tree-based pseudonyms (e.g., Ash, Oak). The cohort encompassed a diverse range of pediatric central nervous system tumors, including medulloblastoma (*n*=6), high-grade glioma and diffuse midline glioma (*n*=6), IDH-mutant astrocytoma (*n*=1), pilocytic astrocytoma (*n*=1), ependymoma (*n*=2), atypical teratoid/rhabdoid tumor (ATRT; *n*=2), choroid plexus carcinoma (*n*=2), embryonal tumor with multilayered rosettes (ETMR; *n*=1), pineoblastoma (*n*=1), and CNS embryonal tumor, NOS (*n*=1) (Figure 1C). Samples from all individuals had undergone prior clinical molecular testing, providing data for concordance assessment. Nineteen samples were sequenced from the same tissue source used for clinical molecular testing, and four samples (Maple, Oak, Ginko, Ipe) were sequenced from autopsy tissue while clinical testing had been performed on surgical resection, biopsy, or CSF specimens.

Samples were sequenced using one or both of two approaches: wgLRS without enrichment, or AS with real-time computational enrichment for a panel of cancer-relevant genes. Of the 23 samples, six (Maple, Oak, Ginko, Aspen, Madrona, Hemlock) underwent both wgLRS and AS from independent library preparations, three (Pine, Ash, Cedar) were evaluated only by wgLRS due to insufficient remaining tissue for additional sequencing, and fourteen were evaluated by AS only.

### Sequencing Performance and Comparison of Sequencing Approaches

Whole-genome LRS achieved a mean depth of coverage of 32.1x (range: 27.7–36.4x) across the nine samples evaluated (Figure 2A, **Supplementary Table 2**). Read N50 ranged from 7.9–19.0 kbp, and median read identity exceeded 99% for all samples. Across 20 AS runs, median on-target autosomal coverage was 112x (range: 62.6–135.9x) with genome-wide background coverage of 9.1–23.7x (Figure 2B, **Supplementary Table 3**). The on-target enrichment enabled high-confidence variant calling in clinically relevant genes while the background genome-wide reads supported CNV profiling across all chromosomes.

Two BED file designs were employed for adaptive sampling: a 723-gene panel (106 Mb target space, 3.4% of the GRCh38 reference) and an 812-gene panel that included additional genes from the UW OncoPlexÔ Cancer Gene Panel (160 Mb target space, 5.2% of the GRCh38 reference) **(Supplementary Table 4, Supplementary Table 5)**. Samples sequenced with the 723-gene panel achieved significantly higher mean autosomal target coverage than those sequenced with the 812-gene panel (121.4x versus 90.9x; Welch’s two-sample *t*-test, *p* = 6.8×10^−4^; Cohen’s *d* = 2.8) (Figure 2C). In a covariate-adjusted linear model including total sequencing yield (Gb) and read N50 as covariates, use of the large panel remained strongly associated with lower autosomal coverage (β = −62.7x, p < 10^−6^), indicating that the observed coverage differences are attributable to the larger genomic footprint of the expanded panel rather than run-to-run variation in sequencing performance.

Six samples underwent both wgLRS and AS from independent library preparations, enabling direct comparison of the two approaches. Both methods achieved concordant results for all clinically reported variants in these samples, suggesting that AS provides comparable diagnostic utility to wgLRS. For SNV and indel detection, AS achieved substantially higher depth of coverage in targeted genes (median 106x versus 32x for wgLRS), which may improve sensitivity for low-frequency somatic variants within the target regions. However, AS cannot reliably detect variants outside the defined target space, representing a trade-off against the unbiased variant detection of wgLRS. The off-target background reads generated during AS were sufficient for analysis by both QDNAseq and ichorCNA, thus both wgLRS and AS provided adequate genome-wide coverage for CNV profiling. Tumor purity estimates derived from the two approaches were also highly concordant (*r* = 0.95, mean absolute difference 0.034), indicating that the sparse genome-wide background generated during AS is sufficient for purity estimation. For methylation classification, both approaches provided adequate CpG coverage for classifier performance, with no systematic difference in classification accuracy between wgLRS and AS.

### Concordance of Single Nucleotide Variant and Indel Detection

Clinical molecular testing reported 51 SNVs or indels in 17 of 23 samples and included both somatic and germline variants **(Supplementary Table 6)**. These included canonical driver mutations in *TP53*, *H3–3A* (K27M; note that clinical testing often identifies this variant as p.K28M, and we use both interchangeably here), *CTNNB1*, *IDH1*, *BRAF* V600E, and other genes, as well as frameshift and nonsense variants in tumor suppressors including *ARID1A*, *PTCH1*, *BAP1*, *SMARCB1*, and *RB1* (Figure 3A). Using LRS, we detected 42 of 51 variants (82%), with no difference between AS and wgLRS. Concordance was strongly influenced by variant allele fraction (VAF) and the variant calling pipeline employed. All but one of the variants present at VAF ≥15% were detected by both wgLRS and AS.

Somatic variant calling was performed using both Clair3 (a germline-oriented caller) and ClairS-TO (a somatic-tumor-only variant caller optimized for ONT data). ClairS-TO called four clinically reported variants not called by the standard Clair3 pipeline **(Supplementary Table 6)**: *H3–3A* p.K28M in Pear, *IDH1* p.R132L and *TP53* p.R158H in Cherry, and *TP53* p.G108Vfs*15 in Ipe. All four variants were confirmed by manual IGV inspection and represent known driver alterations in their respective tumor types. Notably, the *H3–3A* p.K28M variant in Pear was present at an allele fraction of approximately 0.20 by ClairS-TO (0.24 on manual IGV review), yet was not called by standard Clair3, suggesting that somatic-optimized callers can improve detection of variants at moderate allele fractions in tumor samples.

A subset of low-frequency somatic variants reported by clinical testing were not detected by LRS **(Supplementary Table 6)**. For example, the *SETD2* p.R1459* variant in sample Pine, reported at an unspecified VAF by clinical testing, was visible on only 3 of 24 LRS reads by manual IGV inspection (with one read having low base quality) and was not called by either Clair3 or ClairS-TO **(Supplementary Figure 1)**. Similarly, a *BRAF* p.M668L variant reported at 4% VAF in sample Hemlock was not detected, and manual review confirmed no genomic T>A change at the expected position (Chr7:140,739,937) across any read despite 146x depth of coverage from AS, suggesting either tumor heterogeneity between tissue aliquots or a very low frequency subclonal variant below the LRS detection threshold. Finally, the *PBRM1* p.E927* variant in sample Papaya, reported by clinical testing at 18% VAF, was not detected by LRS or on manual IGV review. This variant was the only variant with VAF ≥ 15% that was not detected by LRS.

### Concordance of Copy Number Variant Detection

Copy number alterations are central to the molecular classification of many pediatric brain tumors, including medulloblastoma subgrouping, identification of high-risk features, and detection of therapeutic targets. Clinical testing reported CNVs in 22 of the 23 samples, ranging from single chromosomal arm gains or losses to complex patterns involving multiple chromosomes and focal amplifications. We evaluated the concordance between LRS and standard clinical testing using CNV profiles generated by both QDNAseq and ichorCNA, with the latter providing tumor purity-corrected copy number estimates (Figure 3B, Figure 3C, **Supplementary Figure 2, Supplementary Table 7, Supplementary Table 8**). Using this approach, the overall concordance between LRS and clinical CNV calling was high, with LRS detecting 123/131 (94%) of clinically reported variants.

Representative examples of concordant CNV detection included isochromosome 17q (loss of 17p with gain of 17q), a hallmark of Group 3 and Group 4 medulloblastoma, which was clinically reported and correctly identified in Pine and Hemlock. *MYC* amplification (8q24) was detected in Aspen, and *MYCN* amplification (2p24) was identified in Oak and Madrona. Madrona, which clinical testing reported had numerous subclonal events, showed concordant detection of whole chromosome gains (Chr4, Chr7) and losses (Chr3, Chr8, Chr10, Chr16, Chr20) as well as focal *CDK4* amplification that were not readily apparent in the ichorCNA output, but were easily seen in the QDNAseq results **(Supplementary Figure 3)**. Complex CNV patterns characteristic of high-grade gliomas were also accurately captured: Coconut showed whole-chromosome gains of Chr1, Chr2, and Chr7 with losses of Chr10 and Chr11, as well as focal *EGFR* and *MYC* amplification; Pear demonstrated focal KIT/PDGFRA amplification at 4q12 alongside multiple chromosomal losses; and Ipe showed *PDGFRA*, *KIT*, and *CDK4* amplifications consistent with the clinical report. Chromosome 22q loss, including the *SMARCB1* locus, was detected in both ATRT samples (Cedar and Fig), consistent with the pathognomonic biallelic inactivation of *SMARCB1* in this tumor type. In Cherry, subclonal loss of distal 14q reported by clinical testing was captured by both QDNAseq and ichorCNA.

LRS provided advantages over clinical CNV testing. Breakpoint resolution was substantially more precise compared to clinical panel-based testing, which typically reports CNV boundaries only at gene-level resolution. Additionally, LRS identified CNV events not reported by clinical testing. For example, copy number losses (Chr21 and Chr22) were detected in sample Ash, and multiple copy number gains (portions of Chr3q, Chr4, Chr5, Chr15, Chr16, Chr20, Chr21) were noted in sample Papaya. These may represent true positives that fell below clinical reporting thresholds, events outside the targeted regions of panel-based assays, or further evidence of tumor heterogeneity in our sample set.

In 6/23 samples, one or more CNVs reported clinically were not detected or clearly visualized in LRS data. For example, multi-focal, multi-copy gains of Chr7 reported in Ginko were not captured by QDNAseq or clearly visible on ichorCNA plots for either WGS or AS data. A low-level gain of Chr14 in Ash was not evident on the LRS CNV profile. In Hemlock, gain of Chr17 was not clearly seen on the ichorCNA comparison plot for WGS data, though QDNAseq did detect focal duplications in the AS data, suggesting that the two CNV calling approaches have partially complementary sensitivity. These discordances may reflect challenges in detecting subtle copy number changes, particularly in samples with complex ploidy or low tumor purity. Notably, the rejected AS reads are generally shorter (700–900 bp), which is more similar to the short-read sequencing data that both QDNAseq and ichorCNA were designed for. The copy-neutral loss of heterozygosity (cnLOH) of distal Chr 9q reported in sample Fir was verified with manual inspection of targeted regions on 9q which showed stretches of homozygosity consistent with the clinical finding.

ichorCNA provided tumor purity estimates for all LRS experiments. Estimated purity showed no significant correlation with pathologist-estimated tumor content from clinical testing (Spearman *ρ* = 0.23, *p* = 0.53, *n* = 10) and was systematically lower (mean 0.66 vs. 0.83). Four samples (Apple, Cedar, Cherry, Chestnut) returned degenerate solutions with an estimated purity of 0% despite tumor content of ≥80% by pathology in three of them. These four samples had the lowest copy number burden in the cohort. Of the 18 samples with two or more clinically reported CNVs, all yielded a non-zero purity estimate, whereas four of the five samples with one or zero clinically reported CNVs did not (Fisher’s exact *p* = 0.0006). This is consistent with the dependence of ichorCNA on copy number deviation to infer tumor purity. For the six samples sequenced by both wgLRS and AS, purity estimates derived were highly concordant (*r* = 0.95, mean absolute difference 0.034, maximum 0.088), indicating that the sparse genome-wide background generated during AS is sufficient for purity estimation **(Supplementary Table 8)**.

### Concordance of Structural Variant and Gene Fusion Detection

SVs, including gene fusions, are defining alterations for several pediatric brain tumor entities. Clinical testing identified six gene fusions among five of the samples but only five fusions (83%) were detected by LRS **(Supplementary Table 9)**. Of these, one, the KIAA1549::BRAF fusion in Chestnut, was a simple two-breakpoint inversion; the remainder were more complex changes involving three or more breakpoints that were manually resolved (Figure 3D, **Supplementary Figure 4**). These four complex rearrangements included the ZNF708::RB1 fusion in Avocado, which involved three breakpoints including a translocation between *ZNF708* and *RB1* with a subsequent deletion that included both *RB1* and *CYSLTR2*. The ZFTA::RELA fusion in Apple, which is diagnostic of supratentorial ependymoma, involved 16 breakpoints affecting 5 genes and exhibiting a combination of inversions and intrachromosomal translocation events. Although this was an AS run and some of the breakpoints were outside the target regions, the off-target background reads were sufficient to enable us to reconstruct the complete structure of Chr11 using IGV. Finally, Maple was reported to carry two fusion events, LAMA2::ARID1B and FANCE::ARID1B, both involving genes on Chr6. Manual inspection of each breakpoint revealed multiple rearrangements involving all three genes, and evaluation of INV and BND events in the Sniffles2 VCF identified >100 events on Chr6, suggesting that a complex chromothripsis-like event led to the formation of these fusions.

One fusion reported clinically was not identified by LRS. The GSAP::KMT2C rearrangement in Ginko was not called by Sniffles2 and could not be confirmed by manual IGV inspection. The sample used for LRS was obtained at autopsy, while clinical testing had been performed on a primary surgical specimen, and coverage at the relevant loci was moderate (32x WGS, 63x AS). Thus, this discordance most likely reflects tumor heterogeneity between the primary and autopsy specimens rather than a systematic limitation of LRS-based fusion detection.

### Methylation-Based Tumor Classification

A unique capability of ONT sequencing is the ability to perform real-time methylation analysis, enabling tumor classification to begin within minutes of sequencing initiation. Methylation classification was evaluated using three classifiers: Sturgeon, Rapid-CNS^2^, and nanoDx. Tumor classification was assessed at three time points to assess how accurately the classifiers identified the tumor: 10 minutes (Sturgeon only), 1 hour (all three classifiers), and the 72-hour endpoint of the sequencing run (all three classifiers) **(Supplementary Table 10, Supplementary Table 11)**. A pathology report from time of diagnosis or autopsy was available for all samples, and methylation array data were available for 13/23 samples. For one sample, Maple, the pathology report specified only a CNS embryonal tumor NOS without methylation classification detail and was thus excluded from methylation concordance analysis.

After 10 minutes of sequencing (corresponding to approximately 0.06–0.14x genome-wide depth of coverage), Sturgeon had reached a classification concordant with the clinical diagnosis in 18/22 evaluable samples. At both the 1-hour and 72-hour timepoints, 19/22 (86%) were correctly classified by at least two of the three classifiers. At endpoint, 16/19 (84%) of these concordant samples had scores above the developer-defined confidence threshold of 0.95 for Sturgeon (note that Madrona was above 0.95 for wgLRS but below for AS, and is counted as below the threshold here), 17/18 (94%) concordant samples were above the threshold of 90 for Rapid-CNS^2^, and 19/19 (100%) exceeded the nanoDx threshold of 0.15 (Figure 4A). At the 1-hour timepoint, Sturgeon scores were above threshold for 13/19 (68%), reflecting increasing classifier confidence with additional CpG coverage.

Concordance was observed across tumor types, including medulloblastoma subtypes (Group 3, Group 4, WNT-activated), diffuse midline glioma H3 K27-altered, ependymoma (ZFTA::RELA fusion-positive and myxopapillary subtypes), ATRT (SHH and MYC subtypes), choroid plexus carcinoma, IDH-mutant astrocytoma, pilocytic astrocytoma, and pineoblastoma. Notably, the low-grade tumors in the cohort, including Chestnut (pilocytic astrocytoma) and Orange (myxopapillary ependymoma), were correctly classified by all three tools, demonstrating that LRS-based methylation classification is not limited to high-grade malignancies.

Three samples (Pear, Ipe, and Coconut) were discordant across the three classifiers at each time point. Pear showed inflammation-predominant signatures across all three classifiers, with Sturgeon calling it as reactive tissue and nanoDx as inflammatory, rather than matching the clinical diagnosis of diffuse midline glioma H3 K27-altered. This pattern likely reflects low tumor purity or sampling of peri-tumoral tissue with a substantial inflammatory component. Ipe was sequenced from autopsy tissue and was classified as GBM MYCN rather than matching the clinical diagnosis of diffuse pediatric-type high-grade glioma, H3-wildtype, IDH-wildtype. This discordance may reflect post-mortem changes affecting methylation patterns, sampling of non-representative tumor regions at autopsy, or biological overlap between these entities. Coconut was classified by clinical methylation array as DMG H3 K27-altered, EGFR-altered subtype while our classifiers categorized it as a glioblastoma subtype (GBM MYCN by Sturgeon and nanoDx; GBM RTK II by Rapid-CNS^2^). This is likely because all three LRS-based classifiers were trained using the Capper *et al*. reference dataset of 2,801 samples across 82 tumor types, which did not include the EGFR-altered subtype of DMG H3 K27.

Finally, we retrospectively assessed the ability of Sturgeon to classify samples in real time. Ginko was correctly classified as choroid plexus carcinoma at 10 minutes by both wgLRS (Figure 4B) and AS (Figure 4C), and this classification was maintained through the end of sequencing, although the wgLRS confidence score fluctuated below the 0.95 threshold at intermediate timepoints as additional CpGs were sampled. Two runs changed classification between 10 minutes and 1 hour: Madrona sequenced by wgLRS, which initially called Group 3 medulloblastoma before reaching the correct Group 4 classification, and Papaya, which moved from an inflammatory control class to the correct PXA classification. In both cases the 10-minute score was below the developer-defined threshold of 0.95 (0.81 and 0.72, respectively), indicating that these early predictions were not reported with high confidence. Notably, Madrona sequenced by AS reached the correct Group 4 classification at 10 minutes, and no sample was confidently misclassified at any timepoint by Sturgeon. There was no difference in overall classification accuracy between AS and wgLRS at either the 1-hour or 72-hour timepoint (Table 2, **Supplementary Table 10, Supplementary Table 11**).

### Effect of an Expanded Reference Dataset on Classification

To evaluate whether an expanded reference dataset could resolve cases of classifier discordance, we additionally classified all samples using MNP-Flex, which utilizes the DKFZ Brain Tumor Classifier v12.8 reference set encompassing 184 methylation classes. MNP-Flex reported no prediction for 4 of 23 samples (17%) and correct classifications for 17 of the remaining 18 samples at endpoint (excluding Maple), including subclass-level resolution not available from the other classifiers **(Supplementary Table 12)**. The only discordant sample was Ginko, a choroid plexus carcinoma classified by Sturgeon, Rapid-CNS^2^, and nanoDx as a choroid plexus carcinoma, subtype B, while MNP_Flex classified it as subtype A. Among the 17 concordant samples the MNP_Flex score was below the 0.90 threshold used for array-derived methylation calls for 7 samples (41%), but below a recommended LRS threshold of 0.30 for only 3 samples (18%).^16^

MNP-Flex also provided clinically relevant subclass-level resolution for medulloblastoma samples, including Group 4 Subclass V for Madrona (concordant with the clinical Nationwide Children’s methylation array), Group 4 Subclass VIII for Pine, and Group 3 Subclass II for both Ash and Aspen. For sample Peach, MNP-Flex classified the tumor as Pineoblastoma-RB1-altered, consistent with the germline RB1 p.R445* variant detected by both clinical testing and LRS. This level of subtyping is increasingly relevant for risk stratification and treatment planning in pediatric neuro-oncology.

However, MNP-Flex returned “no significant predictions above threshold” for four samples: Pear, Plum, Cherry, and Ipe (all samples sequenced by AS). For Pear and Ipe, this is consistent with the discordant results from the other classifiers and may reflect low tumor purity (Pear) or post-mortem effects (Ipe). For Plum (DMG K27) and Cherry (IDH-mutant astrocytoma), the other three classifiers achieved correct classification, suggesting that the expanded classifier space of MNP-Flex may require higher data quality or CpG coverage for confident prediction in some cases. Notably, for Hemlock, MNP-Flex assigned slightly different subclasses from the WGS (Group 3 Subclass IV) and AS (Group 3 Subclass III) datasets, highlighting that subclass-level assignments may be less stable than class-level predictions.

### Summary of Concordance

Overall, LRS demonstrated high concordance with clinical molecular testing (Table 3). Of 23 samples, 21 (91%) showed concordance across all evaluable variant categories. For methylation classification, 19 of 22 evaluable samples (86%) were correctly classified by the three real-time classifiers, in every case within the first hour of sequencing; adding MNP-Flex at endpoint resolved one additional sample, for an overall methylation concordance of 20 of 22 (91%). For SNV and indel detection, all but one variant with a clinically reported VAF ≥15% were identified, indicating reduced sensitivity for lower-frequency variants or heterogeneity between the samples used for clinical testing and those used for research. The use of a somatic-optimized caller (ClairS-TO) recovered four additional clinically significant driver mutations not reported by the standard germline calling pipeline.

Four samples were sequenced from autopsy tissue while clinical testing had been performed on surgical resection, stereotactic biopsy or CSF specimens (Maple, Oak, Ginko, Ipe). Despite the expected challenges of cross-sample concordance due to spatial and temporal tumor heterogeneity, three of four autopsy cases showed substantial concordance across all variant classes. Maple demonstrated concordant CNV profiles, SNV detection (*KRAS* p.K117N, *ARID1A* p.P703Rfs*113), and gene fusion identification (LAMA2::ARID1B, FANCE::ARID1B). Oak showed concordant CNV findings and ETMR methylation classification. Ginko showed concordant SNVs (*BAP1*, *MN1*, *MSH6*) and choroid plexus carcinoma methylation classification.

Only Ipe showed substantial discordance, driven by methylation classification of a glioblastoma MYCN subtype rather than matching the clinical diagnosis of diffuse pediatric-type HGG; both clinically reported SNVs (*TP53* p.G108Vfs*15 and *NF1* p.S1399*) were detected.

The two non-autopsy samples with notable discordance (Pear, Coconut) had specific biological explanations for the observed differences. Pear showed inflammation-predominant methylation signatures suggesting sampling of peri-tumoral tissue, and Coconut harbored an EGFR-altered DMG subtype not represented in the classifier training data. When same-tissue concordance is considered separately, 18 of 19 (95%) same-tissue samples showed overall concordance, compared with 3 of 4 (75%) different-tissue (autopsy) samples.

## DISCUSSION

### IRB Approval

This study was approved by the Seattle Children’s Hospital Institutional Review Board (protocol STUDY00000945). Samples used in this study were obtained from Seattle Children’s Tumor & Tissue Banking & Biology Protocol, a study approved by the Seattle Children’s Hospital Institutional Review Board (PYRO #14449; biobank SCB802, and #0945; use of biobank samples, SCB909). Tissue was collected and used for research under approved protocols with appropriate informed consent.

### Study Design and Sample Collection

We performed a concordance study of 23 pediatric brain tumor samples prospectively collected between January 2024 and July 2025 at Seattle Children’s Hospital (Table 1, **Supplementary Table 1**). To maintain patient anonymity while preserving narrative clarity, patients were assigned unique, deidentified pseudonyms based on tree species (e.g., Ash, Oak, Birch). Samples included fresh-frozen tissue from surgical resections of individuals aged 0–18 years, as well as samples collected at autopsy. The cohort was selected to represent a spectrum of pediatric brain tumors encountered in clinical practice and included both molecularly well-characterized and diagnostically challenging cases. Tumor types included medulloblastoma (*n*=6), high-grade glioma and diffuse midline glioma (*n*=6), IDH-mutant astrocytoma (*n*=1), pilocytic astrocytoma (*n*=1), ependymoma (*n*=2), atypical teratoid/rhabdoid tumor (*n*=2), choroid plexus carcinoma (*n*=2), embryonal tumor with multilayered rosettes (*n*=1), pineoblastoma (*n*=1), and CNS embryonal tumor, NOS (*n*=1) (Figure 1C).

All samples had undergone prior clinical molecular testing at external reference laboratories or at the University of Washington (UW), providing a reference standard for concordance assessment. Clinical testing platforms included the UW OncoPlexÔ cancer gene panel (tumor-only or tumor/normal) and Nationwide Children’s Hospital platforms, including the Somatic Disease/Germline Comparator Exome, Methylation Array for CNS Tumor Classification, and Solid Tumor Fusion Analysis, with specific tests varying by case.

Nineteen samples were sequenced from the same tissue source used for clinical molecular testing. Four samples (Maple, Oak, Ginko, Ipe) were sequenced from autopsy tissue when clinical testing had been performed on surgical biopsy or cerebrospinal fluid (CSF) specimens.

### DNA Extraction, Library Preparation, and Sequencing

For wgLRS, DNA was extracted from fresh-frozen tumor tissue using the Puregene Kit (QIAGEN). DNA libraries were prepared using the SQK-LSK114 Ligation Sequencing Kit (Oxford Nanopore Technologies) according to the manufacturer’s instructions with the following modifications. The end repair and prep reaction was extended to 30 minutes. Following end prep and prior to the first bead cleanup, 4 μL of thermolabile proteinase K (New England Biolabs, P8111) in 26 μL of Q5 buffer (New England Biolabs, B9027) was added to reduce protein carryover from DNA extraction. The adapter ligation reaction was extended to 1 hour.

For AS, DNA was extracted from approximately 25 mg of fresh-frozen tumor tissue using the QIAamp Fast DNA Tissue Kit (QIAGEN) according to the manufacturer’s instructions. For samples analyzed by both AS and wgLRS, the DNA used for each method was derived from the same tissue sample. Sequencing libraries were prepared using the ONT Ligation Sequencing Kit (SQK-LSK114) with a modified protocol designed to achieve a preparation time of less than 60 minutes. Briefly, 1.0 μg of genomic DNA was sheared using a Covaris g-TUBE (two 30-second spins at 11,000 rpm), followed by DNA repair and end prep using a shortened 7-minute incubation (5 minutes at 20°C and 2 minutes at 65°C). Libraries were purified using 0.4x AMPure XP beads and ligated to ONT sequencing adapters at room temperature for 10 minutes, followed by a second bead-based cleanup. Libraries were eluted in 35 μL of elution buffer and quantified using the Qubit dsDNA High Sensitivity assay.

Libraries were loaded onto R10.4.1 PromethION flow cells (FLO-PRO114M) and sequenced for at least 72 hours. To achieve sufficient depth of coverage, some samples required multiple flow cells (range: 1–2 for wgLRS, 1 for AS) and/or multiple library loads per flow cell (range: 1–7 for wgLRS, 1–4 for AS) due to performance issues such as lower-than-expected pore counts and/or sequencing run errors (Figure 2, **Supplementary Table 2, Supplementary Table 3**).

For targeted sequencing experiments, AS was performed using the ONT MinKNOW software to enrich for genes relevant to pediatric brain tumors. Two target BED files were employed: one comprising 723 genes, and a second that incorporated an additional 89 genes from the UW OncoPlexÔ Cancer Gene Panel, expanding the panel to a total of 812 genes (Figure 2, **Supplementary Table 4, Supplementary Table 5**). In both designs, target regions included a 20-kb buffer flanking each gene, covering approximately 106 Mb or 160 Mb of genomic space, respectively. During AS, reads aligning to target regions were sequenced to completion, while off-target reads were ejected from the pore after partial sequencing. The resulting off-target reads, though short (median N50 ~700–800 bp), provided low-depth genome-wide coverage sufficient for copy number analysis.

### Basecalling and Alignment

Raw Nanopore signal data (POD5 format) were basecalled using Dorado (v0.9.0 or v1.1.1) using the super-accurate model (dna_r10.4.1_e8.2_400bps_sup@v5.0.0 or dna_r10.4.1_e8.2_400bps_sup@v5.2.0) and the --5hmCG_5mCG modification calling flag to identify 5-methylcytosine and 5-hydroxymethylcytosine modifications at CpG sites. Basecalled reads were aligned to GRCh38 using minimap2 (v2.28)^18^ with the - ax map-ont preset. Aligned reads were sorted and indexed using samtools (v1.19).^19^

### Single Nucleotide Variant Calling

Single nucleotide variants (SNVs) and small insertions/deletions (indels) were identified from the aligned BAM files using two complementary approaches. Germline variant calling was performed using Clair3 (v1.0.8)^20^ with the r1041_e82_400bps_sup_v500 model. The resulting VCF file was phased using WhatsHap (v1.7).^21^

Somatic variant calling was performed using ClairS-TO (v0.4.2),^22^ a somatic-tumor-only variant caller optimized for ONT data that does not use a matched normal sample. ClairS-TO was run on the same aligned BAM files using the platform parameter flag ‘platform=ont_R10_dorado_sup_5khz_ssrs’ with the output limited to canonical chromosomes. Variants called uniquely by ClairS-TO (i.e., not present in the standard Clair3 output) were identified by comparing the ClairS-TO output to the Clair3 output using a custom script to identify variants only called by ClairS-TO (Figure 3A, **Supplementary Figure 1, Supplementary Table 6**).

Variants identified by Clair3 were annotated using Ensembl Variant Effect Predictor (VEP, v114),^23^ incorporating the following databases and prediction tools: AlphaMissense, CADD, SpliceAI, gnomAD, ClinVar, Enformer, OpenTargets, SpliceVault, and Dosage Sensitivity.^24–31^ Annotated variants were filtered to retain only those with a population allele frequency below 1% in both gnomAD and the 1000 genomes variant database. Somatic variants called by ClairS-TO were annotated for oncogenicity using OncoKB to identify known or likely oncogenic alterations and potential therapeutic targets.^32^

Clinically reported variants were compared against LRS calls by manual review. Variants not called by either pipeline were evaluated by manual inspection in the Integrative Genomics Viewer (IGV) to determine whether supporting reads were present below the caller’s detection threshold.^33,34^

### Copy Number Variant Calling

Copy number variants (CNVs) were identified from the aligned BAM files using QDNAseq (v1.42.0)^35^ with pre-computed GRCh38 bin annotations at 100 kb resolution **(Supplementary Table 7)**^36^. In addition, tumor purity-corrected copy number estimates were generated using ichorCNA (v0.4.0) using a bin size of 1 Mbp **(Supplementary Table 8)**.^37^ ichorCNA was run without a user-supplied purity estimate, allowing tumor fraction to be inferred de novo from the copy number data. Copy number segments were annotated for overlap with RefSeq genomic features using BEDTools (v2.31.1).^38^ CNV concordance with clinical testing was assessed by comparing whole-chromosome and arm-level events, focal amplifications, and homozygous deletions **(Supplementary Figure 2)**. Tumor purity estimates from ichorCNA were compared with the tumor content reported by clinical molecular testing, where available (*n*=13). For samples returning multiple candidate solutions, the highest ranked maximum-likelihood solution was used, and solutions returning an estimated purity of zero were treated as non-informative.

### Identification of Structural Variants and Gene Fusions

SVs were called from the aligned reads using Sniffles2 (v2.7.0)^39^. Sniffles2 calls were phased using LongPhase (v1.7.3).^40^ Gene fusions reported by clinical testing were evaluated in LRS data by reviewing Sniffles2 SV calls for interchromosomal translocations (BND events) and intrachromosomal rearrangements involving the relevant gene loci. We determined population frequency for SVs using a reference panel of 500 individuals from the 1000 Genomes Project previously sequenced on the ONT platform with needLR (v 3.5).^41,42^ Candidate fusions were confirmed by manual inspection in IGV, evaluating the number of supporting chimeric reads and the breakpoint positions relative to known gene structures (Figure 3, **Supplementary Figure 2, Supplementary Table 9**).

### Methylation Classification

Methylation-based tumor classification was performed using three independent tools: Sturgeon (v0.5.0)^12^, the methylation classification module of Rapid-CNS^2^ (v0.3)^11^, and nanoDx (v 0.1.0)^13^ (Figure 4, Table 2, **Supplementary Table 10**). All three classifiers were trained on or derived from the reference dataset described by Capper *et al*., which comprises DNA methylation profiles from 2,801 central nervous system tumor and reference tissue samples spanning 82 methylation classes.^3^ For Sturgeon, raw base modification probabilities in the aligned BAM files were standardized to canonical format and extracted using modkit (v0.5.0, ONT). Single-read modification calls were mapped onto GRCh38 target array CpG sites using Sturgeon inputtobed and provided to sturgeon for classification. For Rapid-CNS^2^, aligned BAMs were run through the ONT EPI2ME wf-human-variation pipeline to generate bedmethyl files. The samples were evaluated against Rapid-CNS^2^, GRCh38 target arrays leveraging R-mediated Random Forest classification. For nanoDX, DNA methylation was quantified and formatted as bedMethyl files using modkit (modkit pileup). Using BEDTools, these methylation sites were then intersected with targets from the standard Illumina HumanMethylation450K array. Finally, the resulting methylation profiles were analyzed using the NanoDX Neural Network to predict tumor subtypes and their probabilities.

To simulate real-time sequencing progression using Sturgeon aligned BAM files were subsetted by read timestamp using samtools (v1.22)^19^. At each time point, methylation calls were extracted and submitted to the classifier. The earliest time point at which Sturgeon produced a correct classification (concordant with clinical methylation array or pathology diagnosis) was recorded. Sturgeon classifications, confidence scores, aligned read counts, and approximate genome-wide coverage at each time point are reported for all 29 sequencing runs **(Supplementary Table 11)**.

Concordance was evaluated at two key time points: the 1-hour mark (representing a clinically relevant intraoperative window) and the sequencing endpoint. For samples with both wgLRS and AS data, classification was performed independently on each dataset (Table 2, **Supplementary Table 10**). Developer-defined confidence thresholds were used to evaluate classifier performance: a calibrated score ≥0.95 for Sturgeon, a score ≥ 0.9 for Rapid-CNS^2^, and a score ≥0.15 for nanoDx.^11–13^

In addition to these three classifiers, we evaluated MNP-Flex (v1.0),^16^ an updated methylation classifier developed as part of the Rapid-CNS^2^ platform. Unlike Sturgeon, Rapid-CNS^2^, and nanoDx, which were trained on the original Capper *et al*. reference dataset comprising 82 methylation classes, MNP-Flex utilizes the expanded DKFZ Brain Tumor Classifier v12.8 reference set, which encompasses 184 methylation classes including recently defined tumor subtypes. MNP-Flex classification was performed on endpoint sequencing data only (not time-course), using both WGS and AS aligned BAM files **(Supplementary Table 12)**. For MNP-Flex concordance was evaluated for sub-type only, but all classification categories were recorded.

### Concordance Assessment

Concordance between LRS and clinical testing was assessed across four variant categories: SNV/indel detection, CNV detection, SV/gene fusion detection, and methylation classification (Table 3). For each sample and variant category, concordance was classified as follows. Concordant: All clinically reported findings in that category were detected by LRS, or no variants were reported in the clinical report. Partially concordant: The majority of clinically reported findings were detected, with minor discrepancies attributable to known technical limitations (e.g., low-frequency variants below detection threshold, subtle copy number changes) or a difference in the sample used for clinical testing and the one used for this study. Discordant: Clinically reported findings were not detected by LRS, or LRS classification differed substantively from the clinical result. Not evaluable: No variants of that type were reported by clinical testing, or insufficient data for comparison. Overall sample-level concordance was scored as “Concordant” if all evaluable variant categories were concordant or partially concordant, and “Discordant” if any category showed full discordance without a clear biological explanation (e.g., different tissue source).

### Statistical Analysis

Coverage metrics were compared between AS experiments performed using the Large (812) and Small (723) gene target BED files. Autosomal target coverage was treated as a continuous outcome, and differences between groups were assessed using Welch’s two-sample t-test. Effect size was quantified using Cohen’s d. To evaluate whether coverage differences were independent of run-level sequencing characteristics, a covariate-adjusted analysis was performed using multiple linear regression, with autosomal coverage as the outcome, BED file design (larger vs. smaller) as the primary predictor, and total sequencing yield (Gb) and read N50 as covariates. Model assumptions (linearity, normality of residuals, homoscedasticity) were assessed by visual inspection of diagnostic plots. All statistical tests were two-sided, and p < 0.05 was considered statistically significant.

## Supplementary Material

Supplementary Files

This is a list of supplementary files associated with this preprint. Click to download.

- ONTBrainTumorSupplementalData.xlsx
- ONTBrainTumorSupplementalFigures.docx

## Figures and Tables

**Figure 1:**
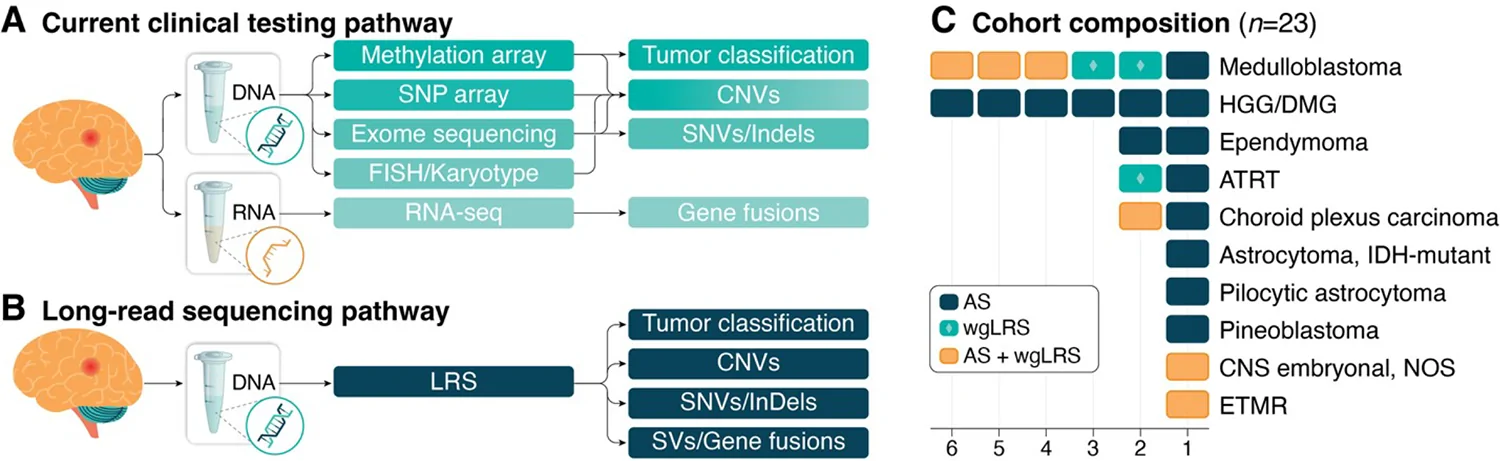
Study design and sample summary. Comparison of **(A)** current multi-platform clinical workflow for molecular characterization of pediatric brain tumors with **(B)** the single-platform long-read sequencing (LRS) approach evaluated in this study. Current workflows require separate assays for SNV/indel detection (targeted panel sequencing), copy number profiling (chromosomal microarray or shallow WGS), gene fusion detection (RNA sequencing or fusion panels), and methylation classification (methylation array), with complete results typically available in 4–8 weeks. LRS-based approaches can capture similar information from a single library preparation and sequencing experiment, with some results available within hours of sequencing starting. Thus, LRS has the potential to dramatically reduce the amount of tissue needed for comprehensive evaluation while dramatically reducing turnaround times. **(C)** Distribution of tumor types in the study cohort and sequencing approaches employed. Six samples underwent both whole-genome LRS (wgLRS) and adaptive sampling (AS) from independent library preparations. CNV, copy number variant; SNV, single nucleotide variant; SV, structural variant.

**Figure 2:**
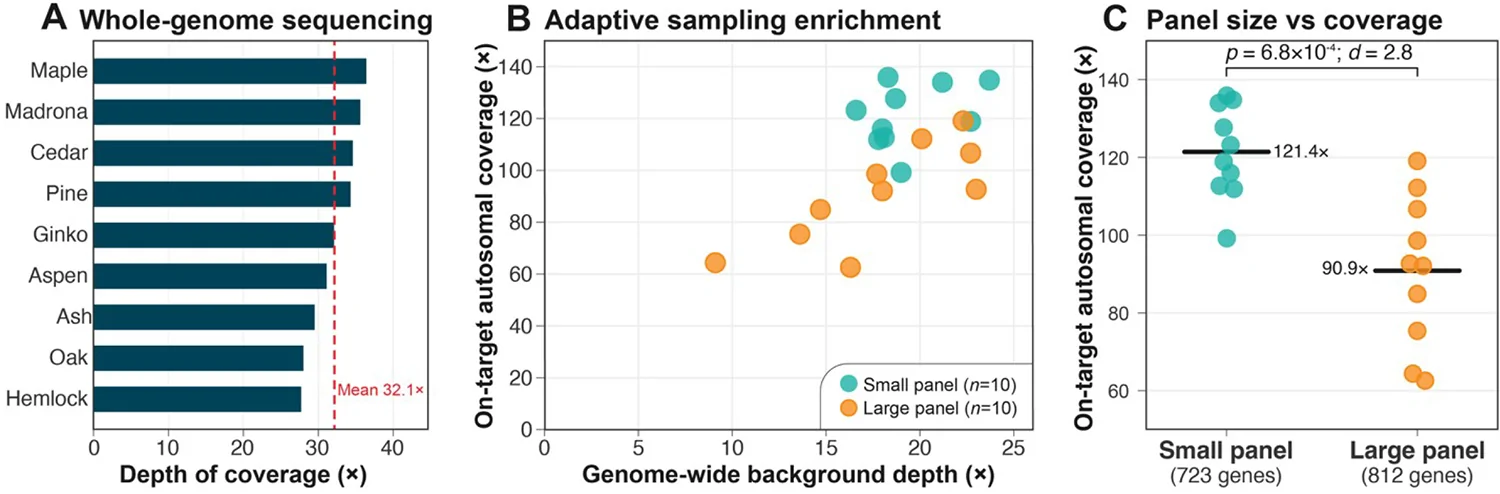
Sequencing performance metrics. **(A)** Depth of coverage for whole-genome long-read sequencing (wgLRS) across nine samples (mean 32.1x; range 27.7–36.4x). **(B)** Genome-wide background coverage and on-target coverage for 20 adaptive sampling runs. BED file designs comprise a smaller 723-gene panel (106 Mb) and a larger 812-gene panel (160 Mb). **(C)** Comparison of on-target autosomal coverage between the two BED file designs. The smaller panel achieved significantly higher per-target coverage attributable to the smaller genomic footprint of the smaller bed file.

**Figure 3:**
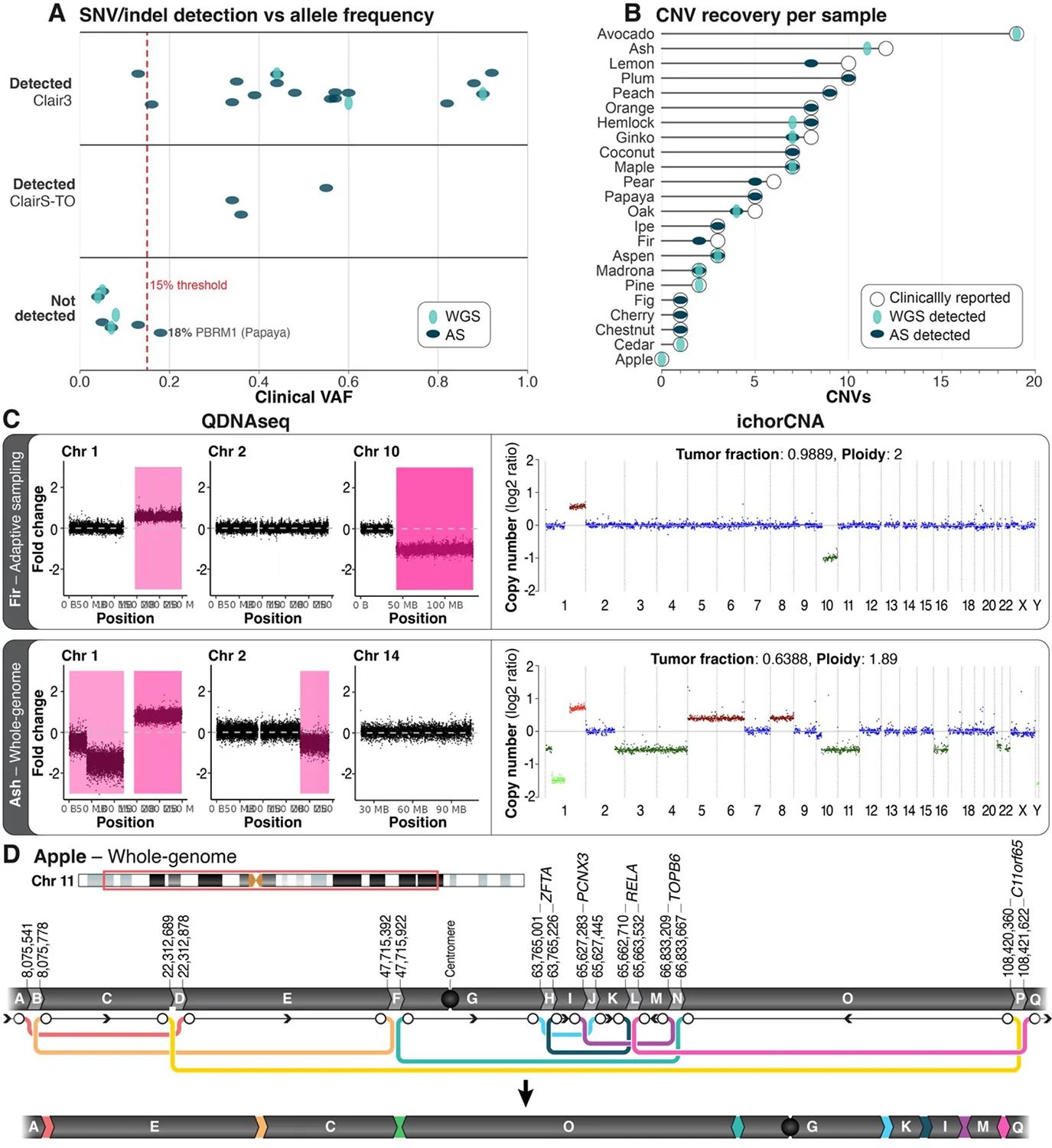
Concordance of variant detection with clinical testing. **(A)** Landscape of 27 clinically reported SNVs and indels for which the VAF was reported by clinical testing, annotated by detection status in LRS (detected by Clair3, recovered only by ClairS-TO, or not detected) and by clinical variant allele frequency. Across the cohort, 24 additional SNVs and indels were clinically reported and are not shown here: 21 detected by Clair3, 1 detected only by ClairS-TO, and 2 not detected. **(B)** Concordance of copy number variant (CNV) calls by sample and sequencing approach. **(C)**Representative copy number profiles identified by LRS for two samples, Fir (AS), and Ash (wgLRS). Plots are generated by QDNAseq and ichorCNA. Only a subset of chromosomes from QDNAseq output are shown. The comparison between the two methods illustrates concordant detection of hallmark events using both WGS and AS (for example gain of 1q and loss of 10q in sample Fir). **(D)**Subway plot of the fully resolved ZFTA::RELA fusion in Apple, reconstructed from AS data, showing 16 breakpoints that bisect five genes on Chr 11.

**Figure 4:**
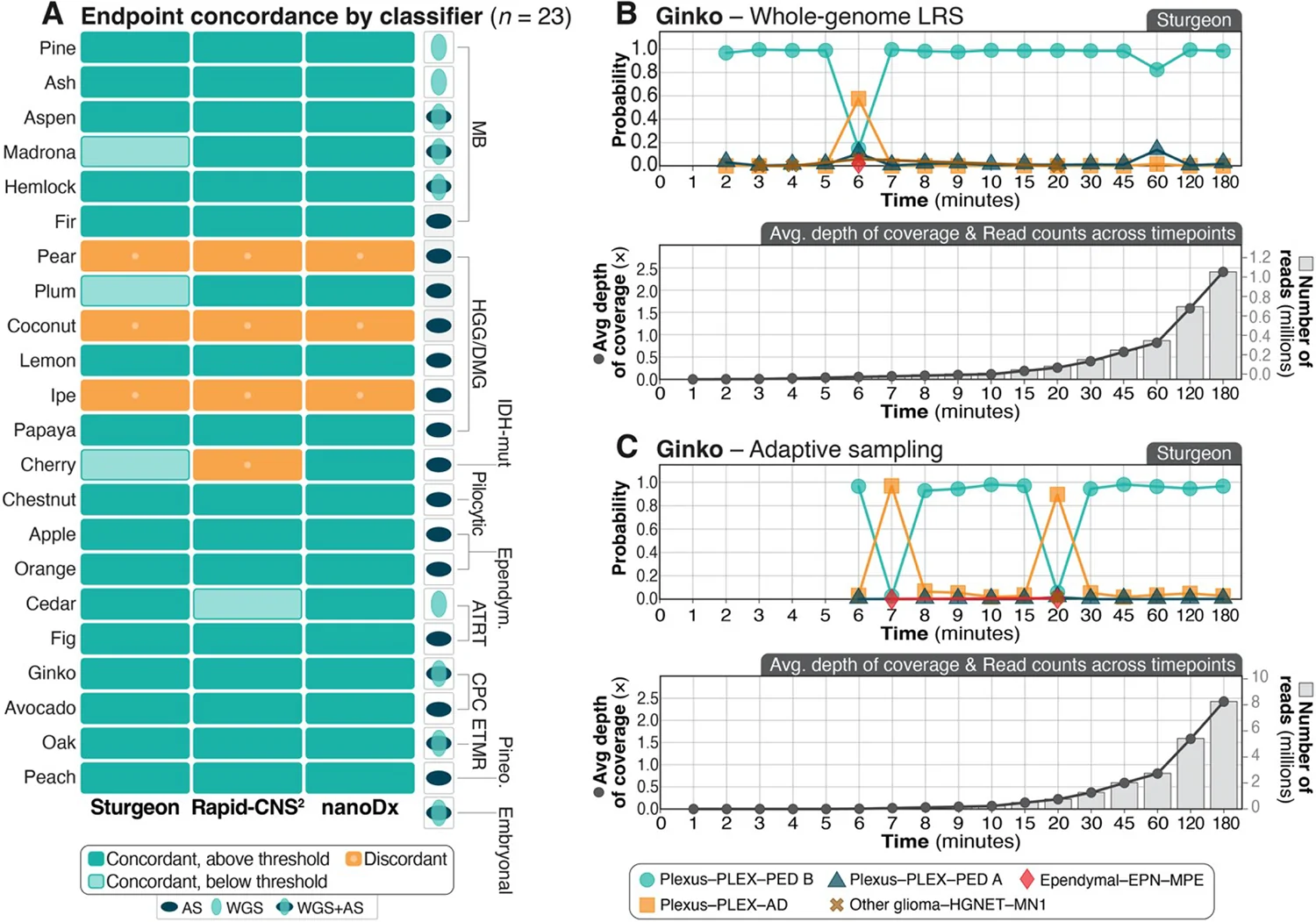
Methylation-based tumor classification. **(A)**Endpoint classification concordance across four methylation classifiers for all 23 samples. Samples are grouped by clinical tumor type. **(B,C)**Performance of Sturgeon over time for wgLRS (**B**) and AS (**C**) of Ginko (Choroid plexus carcinoma) both demonstrate accurate classification after 10 minutes of sequencing.

**Table 1:** Sequencing strategy and diagnoses for the 23-sample study cohort. Tumor classifications follow 2021 WHO CNS tumor nomenclature. Sample source indicates whether tissue was obtained from surgical resection or autopsy.

Sample name	Sequencing method	Sample source (this study)	Diagnosis
Maple	WGS, AS	Autopsy	CNS embryonal, NOS
Oak	WGS, AS	Autopsy	ETMR
Ginko	WGS, AS	Autopsy	Choroid plexus carcinoma
Pine	WGS	Surgical resection	Medulloblastoma, G4
Fir	AS	Surgical resection	Medulloblastoma, WNT
Ash	WGS	Surgical resection	Medulloblastoma, G3
Cedar	WGS	Surgical resection	ATRT
Aspen	WGS, AS	Surgical resection	Medulloblastoma, G3
Madrona	WGS, AS	Surgical resection	Medulloblastoma, G4
Hemlock	WGS, AS	Surgical resection	Medulloblastoma, G3
Apple	AS	Surgical resection	Ependymoma, ZFTA-RELA
Orange	AS	Surgical resection	Myxopapillary ependymoma, spine
Papaya	AS	Surgical resection	PXA
Pear	AS	Surgical resection	DMG, H3 K27M-mutant
Plum	AS	Surgical biopsy	DMG
Chestnut	AS	Surgical resection	LGG - pilocytic astrocytoma
Coconut	AS	Stereotactic biopsy	DMG
Cherry	AS	Surgical resection	DMG, IDH-mutant
Avocado	AS	Surgical resection	Choroid plexus carcinoma
Peach	AS	Surgical resection	Pineoblastoma
Fig	AS	Surgical resection	ATRT
Lemon	AS	Stereotactic biopsy	DMG, H3 K27M-mutant
Ipe	AS	Autopsy	HGG - IDH wildtype, H3 wildtype

WGS, whole genome sequencing; AS, adaptive sampling; ATRT, atypical teratoid/rhabdoid tumor; DMG, diffuse midline glioma; ETMR, embryonal tumor with multilayered rosettes; HGG, high-grade glioma; PXA, pleomorphic xanthoastrocytoma.

**Table 2:** Methylation Classification Results. Comparison of LRS methylation classification with clinical diagnosis for all 23 samples at the end of sequencing. Classifications shown from three classifiers and assessed against clinical methylation array or pathology diagnosis. Results from MNP-Flex are not shown here. Methylation classification was considered concordant if at least two of the three real-time classifiers (Sturgeon, Rapid-CNS^2^, nanoDx) made the concordant diagnosis, regardless of whether the score fell below the developer-defined threshold.

Sample	Clinical Diagnosis	Method	Sturgeon (endpoint)	Rapid-CNS^2^ (endpoint)	NanoDx (endpoint)	Concordant
Oak	ETMR	WGS	ETMR	ETMR	ETMR	Y
		AS	ETMR	ETMR	ETMR	Y
Ginko	PLEX	WGS	PLEX, PED B*	PLEX_PED_B	PLEX, PED B	Y†
		AS	PLEX_PED_B	PLEX_PED_B	PLEX_PED_B	Y†
Pine	MB, G4	WGS	MB, G4	MB_G4	MB, G4	Y
Ash	MB, G3	WGS	MB, G3	MB_G3	MB, G3	Y†
Cedar	ATRT	WGS	ATRT, SHH	ATRT_SHH*	ATRT, SHH	Y
Aspen	MB, G3	WGS	MB, G3	MB_G3	MB, G3	Y
		AS	MB, G3	MB, G3	MB, G3	Y
Madrona	MB, G4	WGS	MB, G4	MB_G4	MB, G4	Y
		AS	MB, G4*	MB_G4	MB, G4	Y
Hemlock	MB, G3	WGS	MB, G3	MB_G3	MB, G3	Y
		AS	MB, G3	MB, G3	MB, G3	Y
Fir	MB, WNT	AS	MB, WNT	MB_WNT	MB, WNT	Y
Apple	EPN, ZFT-ARELA	AS	EPN, RELA	EPN_RELA	EPN, RELA	Y
Orange	MPE	AS	EPN, MPE	EPN_MPE	EPN, MPE	Y
Papaya	HGG / PXA	AS	PXA	PXA	PXA	Y
Pear	DMG, H3 K27M	AS	CONTR, REACT	LGG_PA_PF*	CONTR, INFLAM	N
Plum	DMG, H3 K27-altered	AS	DMG, K27*	DMG_K27	DMG, K27	Y
Chestnut	Pilocytic astrocytoma	AS	LGG, PA PF	LGG_PA_PF	LGG, PA PF	Y
Coconut	DMG, H3 K27-altered	AS	GBM, MYCN*	GBM_RTK_II	GBM, RTK II	N
Cherry	A, IDH-mutant	AS	A IDH*	LGG_DNT*	A IDH	Y
Avocado	PLEX	AS	PLEX, PED B	PLEX_PED_B	PLEX, PED B	Y
Peach	Pineoblastoma	AS	PIN T, PB A	PIN_T_PB_A	PIN T, PB A	Y
Fig	ATRT, MYC subtype	AS	ATRT, MYC	ATRT_MYC	ATRT, MYC	Y
Lemon	DMG, H3 K27-altered	AS	DMG, K27	DMG_K27	DMG, K27	Y
Ipe	HGG, H3/IDH-wildtype	AS	GBM, MYCN*	GBM_RTK_II	GBM, MYCN	N

* The classifier score fell below the developer-defined confidence threshold, see Tables S10 and S11 for per-sample confidence scores.

** Clinical diagnosis was a GBM, which is consistent with embryonal histology, but a specific subtype was not provided thus was excluded from concordance calculation.

† Clinical methylation array not performed. A, astrocytoma; ATRT, atypical teratoid/rhabdoid tumor; DMG, diffuse midline glioma; DPHGG, diffuse pediatric-type high grade glioma; EPN, ependymoma; GBM, glioblastoma; HGG, high-grade glioma; IPA, infratentorial pilocytic astrocytoma; MB, medulloblastoma; MPE, Myxopapillary ependymoma; NP, no prediction; PLEX, choroid plexus tumor; PXA, pleomorphic xanthoastrocytoma.

**Table 3:** Per-sample concordance summary across variant categories. Overall, 21/23 (91%) samples were completely concordant, with discordances in Pear (inflammation signatures) and Ipe (autopsy tissue effects). Considering MNP-Flex results, Methylation was concordant in 20/22 (91%) of samples that could be evaluated, with 18/19 (95%) of samples that were from the same tissue concordant, while 3/4 (75%) from different tissues were concordant. Y, fully concordant; N, discordant; Partial, most findings concordant with minor discrepancies; N/A, clinical testing was not performed for this variant type.

Sample	Sequencing	Same Tissue	Methylation	SNV/Indel	CNV	SV/Fusion	Overall
Pine	WGS	Yes	Y	Partial	Y	N/A	Concordant
Ash	WGS	Yes	Y	Y	Partial	N/A	Concordant
Cedar	WGS	Yes	Y	Partial	Y	N/A	Concordant
Maple	Both	No	N/A	Y	Y	Y	Concordant
Oak	Both	No	Y	Y	Y	N/A	Concordant
Ginko	Both	No	Y	Y	Partial	Partial	Concordant
Aspen	Both	Yes	Y	Y	Y	N/A	Concordant
Madrona	Both	Yes	Y	Y	Y	N/A	Concordant
Hemlock	Both	Yes	Y	Partial	Partial	N/A	Concordant
Fir	AS	Yes	Y	Partial	Partial	N/A	Concordant
Apple	AS	Yes	Y	Y	N/A	Y	Concordant
Orange	AS	Yes	Y	Y	Y	N/A	Concordant
Papaya	AS	Yes	Y	Partial	Y	N/A	Concordant
Pear	AS	Yes	N	Partial	Partial	N/A	Discordant
Plum	AS	Yes	Y	Y	Y	N/A	Concordant
Chestnut	AS	Yes	Y	Y	Y	Y	Concordant
Coconut	AS	Yes	Y	Y	Y	N/A	Concordant*
Cherry	AS	Yes	Y	Y	Y	N/A	Concordant
Avocado	AS	Yes	Y	Y	Y	Y	Concordant
Peach	AS	Yes	Y	Y	Y	N/A	Concordant
Fig	AS	Yes	Y	Y	Y	N/A	Concordant
Lemon	AS	Yes	Y	Y	Y	N/A	Concordant
Ipe	AS	No	N	Y	Y	N/A	Discordant

* Coconut scored as overall concordant because genetic findings (SNV, CNV) were fully concordant and the methylation discordance reflected the absence of the EGFR-altered DMG subtype from the reference set used by the three real-time classifiers and was resolved by MNP-Flex.

## Data Availability

Sequencing data cannot be publicly shared due to IRB restrictions. Summary-level data supporting all analyses are provided in the Supplementary Tables.
